# In Time with the Beat: Entrainment in Patients with Phonological Impairment, Apraxia of Speech, and Parkinson’s Disease

**DOI:** 10.3390/brainsci11111524

**Published:** 2021-11-18

**Authors:** Ingrid Aichert, Katharina Lehner, Simone Falk, Mona Späth, Mona Franke, Wolfram Ziegler

**Affiliations:** 1 Clinical Neuropsychology Research Group, Institute of Phonetics and Speech Processing, Ludwig-Maximilians-Universität München, 80799 Munich, Germany; katharina.lehner@phonetik.uni-muenchen.de (K.L.); Mona.Franke@phonetik.uni-muenchen.de (M.F.); wolfram.ziegler@ekn-muenchen.de (W.Z.); 2 International Laboratory for Brain, Music and Sound Research (BRAMS), Département de Linguistique et de Traduction, Université de Montréal, Montréal, QC H3C 3J7, Canada; simone.falk@umontreal.ca; 3 Neolexon, Limedix GmbH, 80538 Munich, Germany; mona.spaeth@neolexon.de

**Keywords:** apraxia of speech, dysarthria, phonological impairment, perception of rhythm, production of rhythm, auditory cuing, acoustic analysis

## Abstract

In the present study, we investigated if individuals with neurogenic speech sound impairments of three types, Parkinson’s dysarthria, apraxia of speech, and aphasic phonological impairment, accommodate their speech to the natural speech rhythm of an auditory model, and if so, whether the effect is more significant after hearing metrically regular sentences as compared to those with an irregular pattern. This question builds on theories of rhythmic entrainment, assuming that sensorimotor predictions of upcoming events allow humans to synchronize their actions with an external rhythm. To investigate entrainment effects, we conducted a sentence completion task relating participants’ response latencies to the spoken rhythm of the prime heard immediately before. A further research question was if the perceived rhythm interacts with the rhythm of the participants’ own productions, i.e., the trochaic or iambic stress pattern of disyllabic target words. For a control group of healthy speakers, our study revealed evidence for entrainment when trochaic target words were preceded by regularly stressed prime sentences. Persons with Parkinson’s dysarthria showed a pattern similar to that of the healthy individuals. For the patient groups with apraxia of speech and with phonological impairment, considerably longer response latencies with differing patterns were observed. Trochaic target words were initiated with significantly shorter latencies, whereas the metrical regularity of prime sentences had no consistent impact on response latencies and did not interact with the stress pattern of the target words to be produced. The absence of an entrainment in these patients may be explained by the more severe difficulties in initiating speech at all. We discuss the results in terms of clinical implications for diagnostics and therapy in neurogenic speech disorders.

## 1. Introduction

Rhythm characterizes many kinds of human activities. For example, the movement sequences of many physical activities such as swimming, hurdling, or fencing have typical rhythmic structures. In music, rhythm plays a particularly important role as well. In traditional compositions, rhythm is conveyed by a beat structure characterized by a more or less regular sequence of different tone durations and pauses [1]. If a piece of music is based on a regular rhythm, people can dance or clap to the rhythm while listening. Underlying this observation is the ability to entrain body movements to music, i.e., the ability to synchronize with the beat [2,3,4,5,6,7]. It is assumed that children are sensitive to rhythm from an early age [8,9,10].

In all languages, rhythmic structure is a fundamental prosodic feature of spoken utterances. Rhythm in language refers to the alternation of strong and weak elements in spoken words and phrases, which structures the production and perception of utterances in time. The phonetic features that convey rhythm and prosody are duration, loudness, and pitch [11]. In phonology, the rhythmic grouping of a stressed with one or more unstressed syllables is framed in the concept of the metrical foot. Although word stress in English and German can vary across syllable positions, there is a preference for trochaic stress in disyllabic words, i.e., for a metrical foot composed of a strong syllable followed by a weak syllable [12,13,14].

### 1.1. The Role of Rhythm in Speech Production

Models of spoken language production differ in their assumptions regarding the extent to which rhythmic parameters are intertwined with the segmental structure of utterances. Two stages in the speech production process are relevant here: on the one hand, the phonological processing of word forms, and on the other hand, the subsequent phonetic encoding stage where motor programs for speech gestures are retrieved. Access to the phonological form of a word involves the encoding of metrical and segmental information, which is generally viewed as two separate processes. In the speech production model proposed by Levelt and colleagues [15,16], the retrieval of the phonological word form from the mental lexicon involves information about the segmental and metrical structure, which is accessed in parallel, mutually independent stages. The metrical frame of an intended word is assumed to specify the number of syllables and, in cases of irregular stress, the main stress position. For words with regular stress, i.e., when stress assignment is predictable (e.g., the trochaic stress pattern in English or German), the metrical structure is generated by default [17,18].

At the phonetic encoding stage, articulatory-motor programs are retrieved, which then guide speech articulation [19]. The linguistic unit at the center of the phonetic encoding stage in Levelt’s model is the syllable. It is postulated that speakers access a long-term store of gestural scores for the frequently occurring syllables of their language; i.e., they download the motor information for each syllable as a pre-compiled, holistic package [20]. For the production of multisyllabic words and words in phrases, the retrieved syllables are assembled in a linear fashion. In contrast to this account, we have proposed a nonlinear gestural (NLG) model of speech motor planning along lines suggested by articulatory phonology [21]. The NLG model explains the phonetic planning demands for spoken words by a hierarchical architecture, expanding from articulatory gestures over syllable constituents to metrical structures [22,23,24]. The gestural arrangement is nonlinear in the sense that gestures (and the segments they compose) are not planned in a strictly left-to-right sequence; rather, the probability of correctly producing each gesture is a function of its position in the metrical–gestural model of the word as whole. This idea is also in line with the “prosody-first” account, where word form encoding is integrated into an emerging, hierarchically organized prosodic structure [25].

### 1.2. The Influence of Rhythm on Speech Production in Patients with Neurogenic Speech Sound Impairments

In speakers with aphasia, speech production can be affected by a phonological encoding impairment. Usually, patients with aphasic phonological impairments show failures concerning the segmental structure of words. In contrast, the metrical form of words is considered much less vulnerable in this patient group. However, a limited number of aphasic patients were reported who showed metrical errors in naming, repetition, or reading [26,27,28,29].

Apraxia of speech (AOS) is considered an acquired disorder of speech motor programming or phonetic planning [30,31]. The speech production of patients with AOS is characterized by frequent phonetic sound distortions and phonemic errors, as well as by dysfluent and dysprosodic speech [32,33,34]. Until recently, metric influences on the error pattern in patients with AOS were widely neglected. In a study of German speakers with AOS, we directly compared the production of disyllabic trochaic words (e.g., ‘Puma, puma) with disyllabic iambic words (e.g., Ko’pie, copy) [35]. The regular metrical pattern of German (i.e., the trochee) had a facilitating effect on word production in apraxic speakers, with trochees being produced with greater articulatory accuracy and fluency than iambs. This effect was replicated in a more recent study of English-speaking patients with AOS [36]. Therefore, the vulnerability of speech segments to apraxic impairment strongly interacts with word-level prosody—an observation that is compatible with the NLG model described above [37]. 

Dysarthria refers to a heterogenous group of neurogenic speech disorders resulting from paresis of the muscles needed to produce speech, akinesia, ataxia, or dyskinesia. Over and above articulation impairment, patients with dysarthria may also exhibit impairments of speech breathing, voice, and prosody [38]. Rhythmic abnormalities in dysarthric speech may be reflected in altered speech rate, dysfluencies, and reduced stress. In particular, patients with Parkinson’s disease and hypokinetic dysarthria are described with disturbances in prosody [39,40].

### 1.3. The Role of Rhythm in Speech Perception: Theories of Rhythmic Entrainment

The “quasi-rhythmic” nature of spoken language has also been studied in terms of its influences on speech perception. There has long been ample evidence for the relevance of metrical structures to auditory segmentation and parsing processes in language acquisition and adult language processing [41,42,43]. More recently, this evidence has been substantially extended by electrophysiological studies, which hint to the importance of metrical structures for phonological, syntactic, and semantic processing, as well as for monitoring functions and for auditory attentional control [44,45,46,47,48]. For example, rhythmically regular sentence contexts have been found to facilitate the resolution of syntactic ambiguities in auditory sentence processing [49] and the detection of syntactic violations [45,50].

A physiological basis for these effects is assumed to reside in neural entrainment, that is, the phase-locking of oscillatory activity of cortical neuron associations in the auditory cortex to periodicities of an external auditory stimulus [51]. In the case of speech, neural entrainment is hierarchically modulated at different frequency bands reflecting hierarchical temporal modulations in the speech envelope. For example, temporal envelope information pertaining to the syllable level has been found to primarily modulate neural activity in the theta range (4–8 Hz), while delta and beta/gamma bands respond to higher prosodic and segmental information, respectively [51,52]. Neural entrainment is a mechanism that supports the dynamic selection of information in the brain towards a specific goal [53], and thereby underpins speech intelligibility and comprehension during speech perception [51,54]. It is also a fundamental process when aligning motor rhythms to auditory rhythms (“rhythmic entrainment”) [53].

Generally, theories of rhythmic entrainment assume that sensorimotor predictions about upcoming rhythmic structure allow humans to synchronize their actions with the rhythm of external stimuli [5]. For language processing, it is assumed that listening to metrically regular speech establishes rhythmical expectations, which, in turn, might also facilitate speech production processes [3,48]. In other words, regular rhythm in speech provides listeners with a temporal expectancy structure, i.e., the brain extracts rhythmic regularities and sets up expectancies for subsequent production. In everyday conversation, rhythmic entrainment may also play a role, because predictions about another speaker’s utterances for rhythm and rate enable a listener to anticipate the end of a speaker’s turn and thereby facilitate smooth turn taking [55,56].

### 1.4. Rhythmic Auditory Cueing in Patients with Speech Sound Impairments

Speaking to the rhythm of an external timekeeper has a long and successful tradition in the treatment of various speech disorders. For example, the metronome effect was already described in 1969 for the treatment of stuttering. In persons who stutter, coupling speech movements to metronomic beats led to a considerable reduction in stuttering symptoms [57,58]. In the treatment of patients with AOS, different treatment techniques focus on the rhythmic–melodic aspects of speaking [59]. Some of these techniques use internal “pacemakers” (e.g., tapping or counting) [60] or a pacing board [61], while others rely on external cues (e.g., tactile) [62] or metronome pacing [63,64]. Whereas the metronome generates a uniform pace, a metrical pacing therapy developed by Brendel and Ziegler [65] respected the rhythmic properties of natural speech. This study used acoustic pacing signals that were rhythmically adapted to the metrical properties of natural speech. Metrical pacing therapy was found to improve segmental accuracy and fluency of sentence production. Rhythmic stimulation, along with the melodic structuring of speech, is also a central component of melodic intonation therapy (MIT), which has a long tradition in the treatment of patients with aphasia and AOS [66,67,68]. It is assumed that the rhythmic aspect of MIT leads to major improvements in apraxic speakers [69].

Although rhythmic–auditory cueing is also a well-established method in the treatment of dysarthria, the influence of rhythmic cues on speech has rarely been investigated in treatment trials. In persons with dysarthria, rhythmic cues are commonly used as pace setters, with the aim of reducing speech rate and increasing speech intelligibility [70,71,72]. For patients with Parkinson’s disease, it is assumed that dysfunctions at the level of the basal ganglia may impair the internal generation of rhythmic movements, leading to problems with the initiation, generation, and maintenance of speech rhythm [73]. There are numerous studies of these patients that demonstrate the effectiveness of rhythmic auditory stimulation on the (gait) motor function [74]. However, few studies in this patient group addressed the influence of rhythmic stimulation on speech motor function [72]. An auditory priming experiment [75] investigated if the metrical regularity of sentences influences speech motor control in individuals with Parkinson’s disease. In this experiment, participants heard a metrically regular or irregular sentence spoken by a model speaker (prime) and were subsequently requested to read aloud a rhythmically regular or irregular sentence as a response (target). The study showed that speech initiation in individuals with Parkinson’s disease was facilitated by a perceived regular speech rhythm. The authors concluded that regular stress distributions in the speech of an interlocutor allow for enhanced temporal predictions and help persons with dysarthria better accommodate to the speech of their conversational partner.

Following the above mentioned auditory priming experiment with dysarthric speakers [75], we developed a similar paradigm to investigate if articulation of patients with AOS and patients with phonological impairment (PI) also benefit from auditory priming by speech with a regular rhythm [76]. In this study, we conducted a sequential synchronization paradigm based on a sentence completion task. The task required participants to complete a phrase spoken by a model speaker by a disyllabic target word while maintaining the rhythm and fluency of the auditory model. Both patient groups produced fewer segmental errors on target words preceded by regularly stressed prime sentences as compared to prime sentences with a more irregular metrical pattern. They therefore seemed to exploit natural rhythmic cues in heard speech for the segmental realization of the target words. Furthermore, our study confirmed earlier results showing that the symptoms of AOS can be modulated positively by a regular (trochaic) stress of words to be produced [35,36]. The benefit from the regularity of target words could, for the first time, also be demonstrated in individuals with PI. Therefore, there seems to be a robust metrical influence on speech at both the phonetic and the phonological planning stages of speech production.

### 1.5. Aims

In the present study, we asked if individuals with different sound production impairments accommodate their speech to the natural speech rhythm of an auditory model similar to neurotypical speakers (rhythmic entrainment). In addition to patients with AOS and PI, which have already been described in [76], we also studied patients with Parkinson’s disease using the same paradigm to see if the effects spread to lower levels of speech production. To investigate entrainment effects, we related the response latencies of the participants to the spoken rhythm of the prime. The main question was whether accommodation effects, if they occur at all, are more significant after hearing sentences with a metrically regular as compared to an irregular pattern (“Prime effect”). Furthermore, we asked if there is a facilitation effect of regular (trochaic) word stress on speech production in these patients (“Target effect”).

## 2. Materials and Methods

### 2.1. Participants

Thirty-six persons with three types of neurogenic speech sound impairment participated (16 female; mean age 62 years, range 30–83, s.d. = 13.7): twelve patients were diagnosed with apraxia of speech (AOS), all of whom had varying degrees of coexisting aphasia; twelve participants presented aphasic phonological impairments (PI) without AOS; and twelve individuals had hypokinetic dysarthria associated with Parkinson’s disease (PD). All patients were recruited in cooperation with several clinical institutions (see acknowledgements). Furthermore, 24 neurologically healthy speakers (CON) were examined (18 female, mean age 50 years, range 30–70, s.d. = 13.3). All patients and healthy controls were native German speakers.

Patients with PD had a confirmed diagnosis of Parkinson’s disease by a neurologist and a diagnosis of hypokinetic dysarthria determined by a speech therapist. All patients showed typical symptoms of hypokinetic dysarthria, such as reduced loudness, breathy voice, imprecise articulation, reduced stress, or increased speech rate. Motor impairment in PD was described by the Hoehn and Yahr [77] scale (stage I: *n* = 1; stage II: *n* = 3; stage III: *n* = 7; stage IV: *n* = 1). Though we excluded patients with a diagnosis of dementia, eight of twelve patients with PD showed a mild cognitive impairment according to the Montreal Cognitive Assessment [78].

In the aphasic patients, a clear diagnosis of AOS or aphasic phonological output impairment constituted a prerequisite for participation in the study. In these patients, no or only very mild dysarthria was allowed. The diagnosis of AOS was made on the basis of the following criteria: (a) inconsistent occurrence of phonetic distortions; (b) presence of perceived phonemic errors (i.e., well-articulated phoneme substitutions, additions, and deletions); (c) prosodic disturbances such as syllable segregation, phoneme lengthenings, or inadequate pauses; (d) articulatory groping, self-corrections, and effortful speech. Patients with a diagnosis of an aphasic PI primarily exhibited phonological errors, whereas word-level prosody and speech rate were largely intact. All aphasic patients suffered from a left-hemisphere cerebral lesion (19 ischemic, 5 hemorrhagic). According to the profile height of the Aachener Aphasie Test (AAT) [79], all patients had mild to moderate aphasia.

In order to assess functional deficits underlying the repetition and naming abilities, all patients were administered three subtests of the model-based assessment battery LEMO 2.0 (German acronym for Lexikon modellorientiert) [80]: auditory nonword discrimination (36 of 72 items), oral naming (20 items), and auditory word-to-picture matching (20 items; same set of items as in oral naming). In all patients, auditory function assessed by auditory discrimination abilities was considered sufficient to accomplish the sentence completion task administered in this study. Most of the aphasic patients showed some lexical access deficit in spoken naming. However, largely intact word comprehension for the same items, as revealed by the auditory word-to-picture matching task, implied that semantic processing abilities were preserved. Patients with PD showed very good naming abilities; only two patients exhibited performance at threshold. Moreover, auditory word comprehension was intact in all these patients. For a summary of the LEMO 2.0 test results, see Table 1.

To describe the severity and quality of the speech sound impairment at word level, we applied the modified version of the hierarchical word lists, the HWL-compact [81], to all patients (see also Table 1). HWL-compact contains 32 one- to four-syllable picturable words with simple and complex syllable structures. To capture error variability, four selected items are tested multiple times (five times). Word production accuracy was assessed for the presence or absence of phonetic distortions, perceived phonemic errors, and dysfluencies (initiation problems, phoneme lengthenings, pauses, and self-corrections). Regarding word repetition accuracy in the HWL-compact testing, patients with AOS revealed the most severe impairments with the lowest number of correct items for all three error categories. In addition to a high percentage of phonological errors, patients with AOS also showed a considerable number of phonetic errors and word fluency errors in the test. In contrast, patients with PI primarily produced phonemic errors. The occurrence of fluency errors in some PI patients was mainly due to conduite d’approche and self-corrections. The word repetition performance in patients with PD was largely unimpaired in the HWL-compact testing. The presence of consistently hypernasal speech in one patient with PD was not considered in the evaluation.

Though all patients with Parkinson’s had good segmental abilities in word repetition, most of them showed clear impact of the dysarthric impairment on spontaneous speech, where all patients exhibited reduced articulatory precision. Some patients showed additional changes to voice quality, reduced overall loudness, decreased prosodic modulation, and/or an increased speech tempo. Severity of dysarthria was judged by the clinic’s speech language therapists on site (3 mild, 6 moderate, 3 severe).

### 2.2. Materials and Procedure

A sequential synchronization paradigm based on a sentence completion task was conducted. A detailed description of the procedure was made in [76]. The stimulus set contained 96 sentences consisting of five two-syllable words each. The first four words of each sentence served as an auditory prime, while the last word was the target word to be produced by the participant. The prime sentence as well as the target word were controlled for the regularity of the underlying speech rhythm. Half of the prime sentences were metrically regular, comprising a series of four trochaic words (i.e., an alternating strong–weak pattern; Xx Xx Xx Xx). The other half of the prime sentences were metrically irregular, consisting of an alternation of trochaic and iambic words (trochee–iamb–iamb–trochee; Xx xX xX Xx). Target words were either trochees (regular; Xx) or iambs (irregular; xX). There were 48 target words in total. Each target word occurred twice in two different prime conditions, once after a regular prime sentence and once after an irregular prime sentence. All target words were concrete nouns and were low in frequency with regard to spoken/written word frequency (CELEX) [82]. Apart from syllable complexity (i.e., CV and CVC syllables), target words were also controlled for the sound class of the first phoneme.

To ensure that the target words were semantically unpredictable by the preceding prime sentence, a written sentence completion task including 43 healthy participants (28 women, 15 men; mean age: 41.8 years) was conducted. Participants were instructed to complete each prime sentence with the first word they thought of. For only two items, the original wording had to be changed to make the target words less predictable.

Due to the systematic variation of prime sentences and target words, there were four different rhythmic conditions:

(1) Regular prime sentence—trochaic target word (Xx Xx Xx Xx-Xx)
  Example: ’Lena ’pflanzte ’damals ’diese-’Tulpe
    (engl. Lena planted then this-tulip)
(2) Regular prime sentence—iambic target word (Xx Xx Xx Xx-xX)
  Example: ’Friedrich ’neckte ’häufig ’diesen-’Te’nor
    (engl. Friedrich teased often this-tenor)
(3) Irregular prime sentence—trochaic target word (Xx xX xX Xx-Xx)
  Example: ’Jule ver’schenkt je’doch ’diese-’Tulpe
    (engl. Jule gives away though this-tulip)
(4) Irregular prime sentence—iambic target word (Xx xX xX Xx-xX)
  Example: ’Mira be’trog zu’nächst ’diesen-Te’nor
    (engl. Mira cheated at first this-tenor)

The experiment was implemented on a computer where the sentences were presented in a pseudorandomized order. The sequential synchronization paradigm requires participants to complete a phrase spoken by a model speaker by maintaining the rhythm and fluency of the auditory model.

The participants were first familiarized with the target word by hearing a male model speaker producing the whole sentence, including the target word (full sentence model). Immediately after this model, the participants only heard the prime sentence spoken by a female model speaker and were required to complete the sentence fragment with the previously heard target word. Participants were instructed to join in by continuing the speech rhythm of the auditory model. Before starting with the experimental items, the patient was familiarized with the procedure through four practice items, which could be repeated several times.

Prior to the experiment, model stimuli were recorded by two experienced speakers (male and female) using an audio interface (Focusrite Scarlett 2i2) and a Rode NTG-2 microphone. Some of the stimuli of both model speakers were adjusted by minimally shortening or lengthening pauses or segments to ensure that there were no differences in mean speech rate between metrically regular and irregular prime sentences (one-way analysis of variance, F(3, 95) = 0.561, *p* > 0.1).

The experiment was administered using the Software PsychoPy (Psychophysics software v1.90.1) [83]. Stimuli were presented over mobile loudspeakers (Philips multimedia speakers 2.0). New trials were always initiated manually by the experimenter. The participants’ responses were recorded digitally at a sampling rate of 44.1 kHz, using the same technique as for the recording of the model speakers. All participants signed an informed consent form but were naive as to the specific purpose of the study.

### 2.3. Data Analyses

As a primary outcome parameter, response latencies—defined as the time interval between the onset of the last word of the prime sentence and the onset of the spoken reaction—were measured. Latencies were manually marked using Praat [84]. Once the segmentation was completed, a Praat script was used to export the data automatically. A total of 5760 responses were evaluated (96 per participant).

For the acoustic analyses, we excluded responses following articulatory groping (Example: Tenor → p.. t.. t.. tenor) or phonemic searching behavior (Example: Tenor → pen.. ton.. ten.. tenor; *n* = 339) as well as words with phonological errors on the first syllable of the target word (*n* = 329). Furthermore, to a lesser extent, we excluded null reactions (*n* = 70), perseverative errors (i.e., repetition of the preceding target word; *n* = 27), semantic paraphasias (Examples: package → gift, tenor → opera; *n* = 38), and responses where an indefinite or definite article was inserted before the target word (*n* = 17). In 15 trials, the audio recording was missing, or the quality of the recording was too poor for an acoustic analysis due to noise. Latencies of more than 2000 ms (*n* = 32) were also excluded. As a result, a total of 4.893 reactions (84.9%) could be considered in the statistical analyses (i.e., exclusion of 867 reactions).

While a similarly high proportion of reactions had to be excluded in the aphasic subgroups (AOS: *n* = 398, PI: *n* = 410), a significantly smaller proportion of responses were excluded in the patients with PD (*n* = 53). In the healthy controls, only six responses were discarded. There were similar numbers of targets that could not be acoustically analyzed following regular (*n* = 439) and irregular (*n* = 428) prime sentences. However, with regard to the regularity of the target words, more iambic than trochaic words had to be excluded (trochaic: *n* = 331, iambic: *n* = 536).

In order to assess the reliability of the acoustic analyses, 5% of the data were re-analyzed (total: *n* = 288). Equal proportions were selected from the samples produced by the patient groups and the controls (2 patients per group, 36 items per participant). A high correlation between the values measured by the two examiners (first and fifth author) was found (intraclass correlation coefficient, *r* = 0.997, *p* < 0.001).

**Figure 1:**
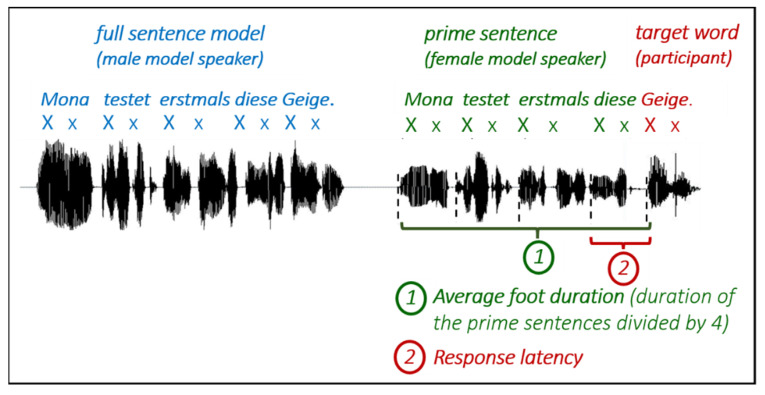
Overview of the procedure and data analysis.

To relate the response latencies to the spoken rhythm of the individual prime sentences, we further determined the average foot duration (i.e., duration of iambs/trochees, in ms) of the model speaker’s prime sentences. For this, the duration of the complete prime sentence was measured and divided by the number of words (i.e., four words per prime). If the participants are sensitive to the temporal predictability of the prime sentences, we expect that they delay their response by the average foot duration of the preceding prime. For an illustration of the procedure, see Figure 1.

### 2.4. Rhythm Discrimination Task

In addition to the sequential synchronization task, all participants performed a rhythm discrimination task (see also [76] for a detailed description of the procedure). The purpose of the task was to ensure that participants were able to perceive the metrical differences between the two priming conditions. Two harmonic tones that differed in duration, volume, and pitch were combined to form a trochaic and iambic tone pair, respectively. Stressed tones were longer in time, higher in pitch, and louder compared to unstressed tones. The trochaic and iambic tone pairs had the same overall duration of 655 ms. Trochaic and iambic tone pairs were then combined to match the rhythmically regular and irregular patterns of the prime sentences in the sentence completion task (regular: Xx Xx Xx Xx, irregular: Xx xX xX Xx). In the experiment, participants heard two tone sequences in succession, separated only by a short pause of 75 ms. They had to decide if the second tone sequence was the same or different from the first tone sequence. Four combinations of regular and irregular tone sequences were created: regular–regular, regular–irregular, irregular–regular, and irregular–irregular. In total, the subjects listened to 32 items (8 items per condition), which were presented in a randomized order. For the analysis, the error numbers were evaluated across the four conditions.

### 2.5. Statistical Analyses

All statistics were performed using R [85]. Linear mixed effects models were calculated to estimate the effects of prime regularity (fixed effects factor PRIME) and target regularity (factor TARGET) on response latencies in the four groups (fixed effects factor GROUP), with ITEMS and SUBJECTS as random slope effects. In several cases, model complexity was reduced after likelihood-ratio testing of a nested vs. the full model using the R-function *ANOVA*.

## 3. Results

### 3.1. Rhythm Discrimination Task

Across all conditions, neurologically healthy individuals made only 6% errors, whereas the patient groups made 20% (PD) and 25% errors (both AOS and PI), respectively. All groups produced considerably fewer errors when the first tone sequence was regular. In these conditions (regular–regular, regular–irregular), none of the healthy speakers made more than one error (out of a total of 16 responses). Comparable performance with no or only one error was also shown by a considerable proportion of patients in all three patient groups (AOS and PI: 8/12 participants, PD: 9/12 participants). Table 2 shows mean error rates for the four rhythmic conditions (regular–regular, regular–irregular, irregular–regular, and irregular–irregular).

The most remarkable result was that error rates were substantially higher when the first stimulus had an irregular rhythm compared to a regular first stimulus, across all groups and regardless of the regularity of stimulus 2.

A generalized linear mixed-effects model was calculated using the function *glmer* of the R-package “lme4” [86]. STIMULUS 1 (regular, irregular), STIMULUS 2 (regular, irregular), and GROUP (CON, PD, AOS, PI) were modeled as fixed effects, and SUBJECT as random intercept effects. The complexity of the full model could be reduced by ignoring the three-way interaction and all two-way interactions with STIMULUS 1, as the variance explained by the simplified model did not differ from that of the full model (likelihood–ratio test using R-function *ANOVA*, χ^2^(7) = 9.0, *p* = 0.25). The simplified model revealed that each of the three patient groups made significantly more errors than the neurotypical controls (AOS: β = 2.07, *p* < 0.001; PI: β = 2.21, *p* < 0.001; PD: β = 1.76, *p* < 0.001), but there were no significant pairwise differences between the three patient samples (*p* > 0.05). A significant increase in the likelihood of a discrimination error was observed across all groups when Stimulus 1 was irregular (β = 1.21, *p* < 0.001). Since there was no interaction of GROUP with STIMULUS 1, the regularity effect of Stimulus 1 was statistically the same for all groups, demonstrating that the participants of all groups were sensitive to the regularity of the first stimulus. Regarding STIMULUS 2, the likelihood of discrimination errors decreased when the stimulus was irregular (β = −0.26, *p* < 0.05), a result that was mainly driven by the large number of errors of the AOS patients when an irregular first stimulus was followed by a regular second stimulus (STIMULUS 2 interactions with AOS: CON: β = 0.39, *p* < 0.05; PI: β = 0.73, *p* < 0.001; PD: β = 0.56, *p* < 0.01), while there were no pairwise differences between CON, PI, and PD regarding the regularity effect of the second stimulus (*p* > 0.05).

### 3.2. Sequential Synchronization Paradigm

**Figure 2:**
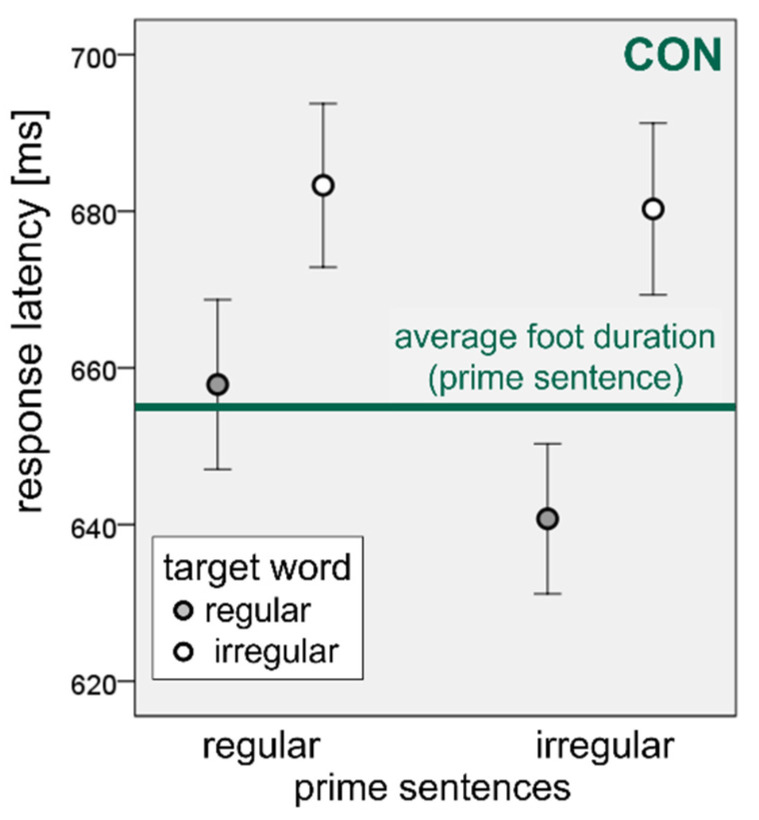
Response latencies (in milliseconds) of the neurotypical participants/CON (mean values, 95% CI of the mean), as a function of the regularity of the prime sentences (abscissa) and the target words (filled and unfilled symbols). The horizontal line represents the grand average of the mean foot durations across all prime sentences.

For the sequential synchronization paradigm, we first present the data for only the healthy controls. Figure 2 plots the absolute onset latencies of the target words, i.e., the distance of the target word onset from the onset of the last word of the prime sentence, as a function of the regularity of the prime and the target for the control group (CON). The horizontal line represents the average foot duration of the prime sentences, which was almost identical for the regular (654 ms) and the irregular (655 ms) primes.

The figure shows that, in the neurotypical controls, responses following regular prime sentences were almost precisely in time with the average prime foot durations when the target word had a trochaic stress pattern. After irregular prime sentences, the trochaic target words had shorter onset latencies. Iambic target words were initiated consistently later, with a delay of ca. 30 ms relative to the “metrical beat”.

A linear mixed effects regression model was calculated for response onset latencies using the function ‘lmer’ of the R-package “lme4” [86]. PRIME (regular, irregular) and TARGET (regular, irregular) were modeled as fixed effects, and SUBJECT as random slope effects. A type III analysis of variance using the function ‘anova’ of the R-package *stats* [85] revealed a significant effect of TARGET (F(1, 23) = 6.8, *p* < 0.05) but not of PRIME (F(1, 23) = 0.3, *p* = 0.59) and a significant PRIME × TARGET interaction (F(1, 2169) = 25.0, *p* < 0.001). The regression coefficients for irregular primes (β = −28.8) and irregular targets (β = 22.6) were both non-significant, while the effect size of the TARGET irregularity in the irregular PRIME condition was highly significant (β = 37.6, *p* < 0.001).

**Figure 3:**
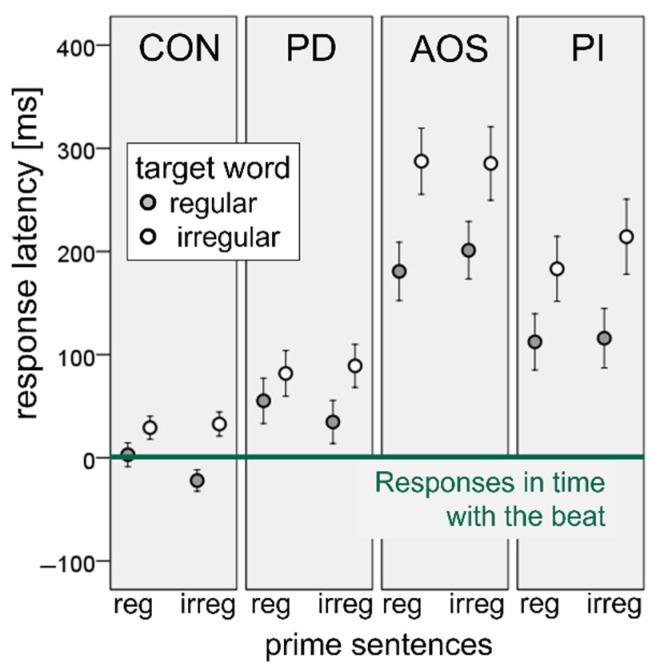
Normalized response latencies (in milliseconds) as a function of prime and target regularity for the healthy control speakers (CON) and the patients with Parkinson’s disease (PD), apraxia of speech (AOS), and phonological impairment (PI). Normalization was performed relative to a predicted latency of exactly the mean duration of the four metrical feet of each individual prime sentence (=0). Error bars are 95% confidence intervals, as in Figure 2.

For the analysis of the entire data set, all response latencies were corrected by the average foot duration of the corresponding prime sentence, so that a response that is exactly in time with the metric beat of the model speaker received a corrected value of 0 (horizontal line in Figure 3). Negative and positive values represent speech initiation latencies shorter or longer than this unit, respectively.

Figure 3 plots these values in milliseconds for each of the four experimental groups, again as a function of the regularity of primes and targets.

The figure illustrates that the CON and the PD group differed only slightly in their response latencies and had similar patterns regarding their sensitivity to the rhythm of the prime sentences and the stress pattern of the target words, whereas the AOS and PI patients had considerably longer onset latencies, which also differed in their sensitivity to the two regularity factors. As with the data of Figure 2, a linear mixed effects model was calculated, this time with GROUP (CON, PD, AOS, PI) as a third fixed effects factor and participants as random slope effects. The complexity of the full model could be reduced by ignoring the three-way interaction, as a likelihood-ratio χ^2^-test revealed that the variances explained by the full and the simplified models were not different (χ^2^(3) = 4.8, *p* = 0.19).

In the simplified model, a type III analysis of variance using the R-function ‘anova’ revealed significant main effects of GROUP (F(3, 59) = 12.2, *p* < 0.001) and TARGET (F(1, 30) = 17.4, *p* < 0.001), but not of PRIME (F(1, 25) = 0.1, *p* = 0.80) and significant interactions of GROUP × PRIME (F(3, 1058) = 2.7, *p* < 0.05), GROUP × TARGET (F(3, 47) = 6.3, *p* < 0.01), as well as PRIME × TARGET (F(1, 4665) = 8.1, *p* < 0.01). The AOS and the PI groups had significantly longer response delays than the healthy speakers (AOS: β = 211.7, *p* < 0.001; PI: β = 161.0, *p* < 0.01) and the Parkinson’s patients (AOS: β = 165.5, *p* < 0.01; PI: β = 105.8, *p* < 0.05), whereas no differences were found between CON and PD (β = 55.7, *p* = 0.25) and between AOS and PI (β = −50.7, *p* = 0.36). Response onset latencies were significantly longer for iambic target words than for trochees (β = 59.9, *p* < 0.05), whereas there was no overall effect of the regularity of PRIME (β = 13.6, *p* = 0.52).

Regarding the two-way effects, the GROUP × PRIME interaction was exclusively driven by a larger effect of irregular vs. regular primes in AOS as compared to CON (β = 34.4, *p* < 0.01), whereas this effect was comparable between all other groups (|β| < 30, *p* > 0.05 in all pairs). The GROUP × TARGET interaction was mainly driven by longer response latencies of iambic relative to trochaic target words in PI compared to CON (β = 71.6, *p* < 0.001) and to PD (β = 71.3, *p* < 0.001), while all other between-group comparisons of the TARGET effect had non-significant regression coefficients (|β| < 40.0, *p* > 0.05).

Since there was an obvious divergence between the healthy speakers and the patients with Parkinson’s dysarthrias, on the one hand, and the two left hemisphere stroke groups on the other hand, further linear mixed effects models were computed for each of these pairs. In the PD vs. CON comparison, the only significant effect was the two-way interaction between PRIME and TARGET that was already seen in the analysis of the CON-data of Figure 2 (β = 37.1, *p* < 0.001). The fact that there was no main effect of and no interaction with GROUP supports that the Parkinson’s patients did not differ from the healthy speakers in their response patterns. In the AOS vs. PI comparison, the only significant effect was an overall increase in onset latencies of iambic vs. trochaic target words across both groups and both priming conditions (β = 79.6, *p* < 0.05), an effect that was also present as a tendency in the CON and PD groups, but did not reach significance there (see Figure 3). All other effects and all interactions were non-significant. In particular, the finding that there was no main effect of and no interaction with GROUP supports that the AOS patients did not differ from the PI patients in their response patterns. Moreover, the absence of a significant prime effect demonstrates that the patients in these two groups were not sensitive to the rhythm of the prime sentence.

## 4. Discussion

The aim of this study was to investigate if individuals with acquired speech sound disorders entrain their speech to the natural speech rhythm of an auditory model similar to neurologically healthy speakers. While interventions based on auditory cueing with rhythmic tone sequences are well-established in the treatment of patients with sound production impairments [59,70], there are only few exploratory studies indicating that these patients might benefit from the rhythm of natural speech input [75,76]. This is the first study to examine rhythmical entrainment in three patient groups with different underlying pathomechanism using the rhythmical priming paradigm described in [76]. In addition to aphasic patients with phonological impairment (PI) or apraxia of speech (AOS), we also studied a group of individuals with hypokinetic dysarthria associated with Parkinson’s disease (PD). Participants were presented with prime sentences with a regular or an irregular rhythmical pattern, respectively, and were instructed to complete them by producing single target words (sequential synchronization task). The target words were also controlled for the regularity of word stress (regular trochaic vs. irregular iambic).

### 4.1. Rhythm Discrimination Task: Were Participants Able to Perceive the Metrical Differences between the Two Priming Conditions?

First of all, we wanted to ensure that participants perceived the rhythmic difference between the regular vs. irregular prime sentences in the sequential synchronization task. For this purpose, we conducted a rhythm discrimination task in which participants had to decide whether there was a difference between two subsequent rhythmic tone sequences. An influence of rhythmic regularity of the first tone sequence was observed in all participants. All groups had much less difficulties in the conditions in which the first stimulus was a regular tone sequence, irrespective of the rhythm of stimulus 2. The healthy speakers and a considerable proportion of the subjects in the patient groups showed no or almost no errors in these conditions. In contrast, all three patient groups had considerable problems when the sentences started with an irregular tone sequence. Therefore, our results show that both the unimpaired subjects and the patients were sensitive to the rhythmical properties of the auditory stimuli.

The advantage of a regular first stimulus in this task is not surprising, considering that participants were able to use the regular rhythm to make perceptual predictions for the processing of incoming inputs. This explanation is in accordance with principles of rhythmic entrainment, where rhythmical expectancies are assumed to be induced by the regular metrical structure of an auditory input [3,48]. Within the framework of the dynamic attending theory [87,88] it is postulated that rhythmically regular tone sequences entrain attentional oscillations and thereby can enhance stimulus processing. Moreover, our results are also consistent with the assumption of an influence of rhythmical stimuli on the capacity of verbal short-term memory. For example, a study from the 1960s already described an advantage of rhythmically grouped items for short-term memory performance [89]. Within the framework of the BUMP model (bottom-up multi-scale population oscillator), Hartley and colleagues explained the relationship between the mechanisms of rhythm perception and the capacity of verbal short-term memory [90]. In their model, sensory processes that are driven by input set up neural oscillations based on the rhythm of speech. In the case of a regular grouping, more oscillators are activated, which then provide a richer and more distinctive representation of the sensory input. Though we used nonverbal tone stimuli in our experiment, it could also be assumed that the rhythmic regular grouping of the first stimulus made the stimulus easier to remember than the irregular grouped stimulus, which, in turn, facilitated the comparison with the rhythmical pattern of the second stimulus.

Especially for dysarthric patients with PD, some authors assume deficits in the auditory processing of temporal cues [91]. For example, in a study by Grahn and Brett, patients with PD were less able to discriminate non-speech rhythms than healthy subjects [92]. This result was explained by deficits in the internal generation of rhythms in this patient group. However, in the experiment performed here, the participants with PD could profit from the regularity of the first stimulus, suggesting that auditory processing can be supported by rhythm (for a discussion of this effect, see Section 4.2). Our results also confirm a former study that revealed that individuals with PD were also able to detect rhythmic differences between tone sequences [75]. The discrepancy between the studies might be explained by the fact that Grahn and Brett [92] used longer and metrically more complex tone sequences in their study.

### 4.2. Sequential Synchronization Paradigm-“Prime Effect”: Do Participants with Neurogenic Speech Sound Impairments Accommodate Their Speech to the Natural Speech Rhythm of an Auditory Model?

The main question was whether accommodation effects, if they occur at all, are larger after hearing sentences with a metrically regular as compared to an irregular pattern. For healthy speakers, our study revealed an entrainment of trochaic target words preceded by regularly stressed prime sentences: participants delayed their response almost exactly by the average foot duration of the preceding prime. In contrast, trochaic target words following irregular prime sentences were produced faster than expected. We assume that, in this condition, a temporal reference frame could not be set up to establish a rhythmic scaffold for the subsequent word production. While hearing a rhythmically irregular prime sentence, the participants may have been unable to create a rhythmical framework for their response and therefore tried to produce the target word as quickly as possible. The results of our study are in line with the assumption that expectancy-induced rhythmic priming can also be used to entrain the speech production of a speaker with the previously heard natural speech signal [3,48]. Although the subjects in our experiment were not involved in a natural conversation, the results also fit into the idea that rhythmic and neural entrainment underlies smooth turn taking processes [55,56]. Importantly, to stay in the rhythm, for the control participants, it was necessary that the target word is also rhythmically regular. Irregular target words were produced slower in both conditions, i.e., an entrainment effect was not evident for these words. In a more general sense, our results confirm a close intertwining of language perception and production processes in neurotypical speakers [93].

Even though somewhat slower across all conditions, individuals with PD showed a pattern similar to that of the healthy speakers. Therefore, dysarthric patients with PD were also able to temporally anticipate and accommodate to the rhythmic regular pattern of an input. This finding confirms our earlier investigation [75], in which patients with PD entrained their speech rhythm especially with another person’s metrically regular speech. Individuals with PD suffer from a progressive loss of dopamine in the basal ganglia, which results in the characteristic motor symptoms of these patients. The basal ganglia are considered to be involved, among other things, in the integration of auditory input into speech motor movements [94]. In particular, the involvement of the basal ganglia in rhythm processing and temporal prediction is widely discussed [95,96,97]. For patients with PD, it was shown that they might benefit from exposure to a temporally predictable, regular auditory cue [98]. Furthermore, numerous studies also revealed that patients with damage to the basal ganglia show improvements in gait function after perceiving rhythmical auditory beats [74,99], suggesting that the patients are still able to access rhythmic entrainment mechanisms to improve their gait patterns. Our results provide further evidence for this assumption within the speech domain and confirm that that these patients are still able to use regular temporal cues to improve their motor impairment, i.e., in our case, their impaired speech production abilities.

In contrast to the dysarthric speakers with PD and the healthy controls, no entrainment could be observed for the aphasic patients with apraxia of speech (AOS) or phonological impairment (PI), respectively. Both patient groups showed considerably longer response latencies and different patterns regarding their sensitivity to the regularity conditions of the experiment. In particular, the regularity of the prime sentences had no influence on their response latencies. A linear model including only these two groups showed no significant main effect of or interaction with the factor PRIME.

The absence of an entrainment in these groups contrasts with the results of our former study, in which we evaluated error production in the same samples of patients with AOS and PI based on the same paradigm and materials [76]. In this former study, both patient groups profited from the metrical regularity of speech input, in that they produced fewer segmental errors on target words preceded by regularly stressed prime sentences as compared to prime sentences with an irregular metrical pattern. Therefore, patients with AOS and PI obviously seemed to exploit rhythmic cues in speech for the segmental realization of the target words. We already discussed this effect within the framework of rhythmic entrainment [3,4,5] and saw the results as further evidence for the assumption that listening to metrically regular speech establish rhythmical expectations, which, in turn, might facilitate speech production processes [48]. The absence of an entrainment effect regarding speech initiation latencies in these patients may be explained by the more severe difficulties in initiating speech at all. If patients take too long to even start producing a word, it is no longer possible for them to stay in the rhythm of the prime sentence.

### 4.3. Sequential Synchronization Paradigm-“Target Effect”: Is There a Facilitation Effect of Regular (Trochaic) Word Stress on Speech Production in Patients with Neurogenic Speech Sound Impairments?

Both the healthy speakers and all three patient groups showed a strong effect of target regularity. Regardless of the regularity of the first sentence, participants initiated irregular (iambic) words with considerably longer latencies compared to regular (trochaic) words. The effect was small in the healthy participants and the Parkinson’s patients and much larger in the two patient groups with AOS or phonological impairment after left hemisphere lesions, and it was independent of the regularity of the prime sentences. For patients with AOS, this result confirms earlier investigations based on repetition tasks, which revealed an advantage of trochaic over iambic patterns [35,36,37]. Furthermore, the results are in agreement with the error data reported from the same experiment for patients with AOS and PI [76]. In both patient groups, the regular metrical pattern of German (i.e., the trochee) had a facilitating effect on word production, with trochees being produced with greater articulatory accuracy and fluency than iambs.

For patients with AOS, the faster initiation of words with trochaic as compared to iambic stress patterns suggests that the encoding of speech segments to apraxic impairment strongly interacts with word-level prosody. Therefore, our study once again supports the assumption of the NLG model that intergestural cohesion expands across the whole metrical tree of a word [22,23,24]. The NLG model assumes a stronger bonding between encoding units within the domain of a trochaic foot (i.e., a stressed unstressed rhythm) than across metrical boundaries, which may explain the facilitating effect of regular words in our patients with AOS. The facilitating effect of trochees in patients with PI could be explained by an entrenchment of segmental articulation in metrical rhythm, as, for example, predicted by the “Prosody First” framework outlined by [25]. It might be that, in patients with PI, the access to nondefault, irregular metrical word forms is more demanding than the spell-out of default, metrically regular forms [27,100].

## 5. Conclusions

Persons with Parkinson’s dysarthria accommodated their speech to the natural speech rhythm of an auditory model similar to neurotypical speakers. Both groups accommodated the initiation of their responses to rhythmically regular speech input (rhythmic entrainment). The sensitivity to the temporal predictability in the individuals with Parkinson’s disease also supports therapeutic approaches based on rhythmic cueing to improve intelligibility in these patients. The absence of an entrainment effect in patients with apraxia of speech and phonological impairment was explained by the more severe difficulties in initiating speech at all, which could be seen in considerably longer response latencies of the target word production. As an additional result, we observed considerably longer latencies for irregular (iambic) words compared to regular (trochaic) words. These acoustic data complemented our previous finding that the stressed–unstressed rhythm promotes accuracy in these patient groups.

## Figures and Tables

**Table 1:** Demographic details and clinical characteristics of the patient sample (12 patients with apraxia of speech, 12 patients with aphasia, 12 dysarthric speakers with Parkinson’s).

Demographics and Test Scores	Apraxia of Speech (AOS; *n* = 12)	Phonological Impairment (PI; *n* = 12)	Hypokinetic Dysarthria (PD; *n* = 12)
Age in years: median (range)	55 (30–78)	60 (41–73)	75 (54–83)
Months post onset: median (range)	63 (2–191)	6 (1–97)	63 (9–213)
Word repetition accuracy ^1^			
Phonetic	44 (2–75)	93 (73–100)	97 (89–100)
Phonemic	64 (19–94)	78 (29–96)	100 (99–100)
Fluency	59 (6–90)	78 (42–98)	99 (94–100)
Auditory word discrimination (LEMO, test V1 ^2^)	10 unimpaired, 1 at threshold, 1 impaired	6 unimpaired, 6 impaired	9 unimpaired, 3 impaired
Auditory word-picture matching (LEMO, test 11 ^2^)	8 unimpaired, 1 at threshold, 3 impaired	7 unimpaired, 3 at threshold, 2 impaired	12 unimpaired
(LEMO, test 11 ^2^)			
(LEMO, test 11 ^2^)			
Verbal naming (LEMO, test 13 ^2,3^)	3 unimpaired, 9 impaired	1 unimpaired, 2 at threshold, 9 impaired	10 unimpaired, 2 at threshold

^1^ Percentage of correct items in the “Hierarchical Word Lists—compact” [81]: Mdn (minimum–maximum), ^2^ Subtests of LEMO 2.0 [80], ^3^ Responses with errors on only one phoneme (e.g., [klait] (engl. dress) → [kait]) were scored as correct.

**Table 2:** Error rates in % (mean, s.d.) in the rhythm discrimination task across the four rhythmic conditions. Figures represent grand averages across patients and items. AOS: apraxia of speech (*n* = 12); PI: phonological impairment (*n* = 12); PD: Parkinson’s dysarthria (*n* = 12); CON: healthy controls (*n* = 24). Eight items per condition.

Stimulus 1	Regular			Irregular		
Stimulus 2	Regular	Irregular	Total	Regular	Irregular	Total
**AOS**	8 (27.8)	3 (17.5)	6 (9.7)	59 (49.4)	29 (45.7)	44 (31.5)
**PI**	5 (22.3)	10 (30.7)	8 (13.2	43 (49.7)	42 (49.6)	42 (25.5)
**PD**	11 (31.9)	6 (23.3)	9 (16.5)	32 (46.8)	33 (47.3)	32 (28.0)
**CON**	1 (11.7)	1 (8.3)	1 (3.5)	14 (34.3)	9 (28.7)	11 (18.4)
**Total**	5 (21.3)	4 (18.4)	4 (10.1)	29 (45.4)	22 (41.2)	25 (27.8)

## Data Availability

The data presented in this study are available on request from the corresponding author. The data are not publicly available due to privacy reasons.
